# Novel Modality of Endoluminal Anastomotic Integrity Assessment with Fluoroangiography After Left-sided Colorectal Resections

**DOI:** 10.1007/s00268-023-06915-8

**Published:** 2023-01-24

**Authors:** Lidia Castagneto-Gissey, Alessandra Iodice, Paolo Urciuoli, Stefano Pontone, Bruno Salvati, Giovanni Casella

**Affiliations:** Department of Surgical Sciences, Sapienza University of Rome, Viale Regina Elena 324, 00161 Rome, Italy

## Abstract

**Background:**

Several methods have been described for the intraoperative evaluation of colorectal anastomotic integrity. Technological evolution has allowed to progress from basic mechanical methods to the use of more sophisticated techniques. This study describes a novel endoluminal modality of colorectal anastomotic assessment through the use of a Disposable Rigid Scope Introducer (DRSI) also allowing for intraoperative endoluminal perfusion evaluation by indocyanine green (ICG) fluoroangiography in patients undergoing left-sided colorectal resection.

**Methods:**

The DRSI consists of an endoluminal introducer device made up of an insertion tube and port connected to an insufflation bulb to manually insufflate the sigmoid and rectum and is compatible with any laparoscopic camera, also allowing for ICG fluoroangiography for perfusion purposes.

**Results:**

The DRSI was successfully used to assess anastomotic integrity after left-sided colorectal resections performed in 16 consecutive patients. The DRSI allowed to visualize by fluoroangiography the quality of tissue perfusion at the anastomotic site in all cases, contributing to the decision of avoiding loop ileostomies in low rectal resections. In 2 cases, the DRSI showed the presence of significant anastomotic bleeding which was successfully controlled by laparoscopic suture placement. No adverse event resulted from the use of this device.

**Conclusions:**

The DRSI combines direct endoluminal visualization of the anastomosis together with real-time evaluation of its blood flow. This device holds great potential for prompt intraoperative detection of anastomotic alterations, possibly reducing the risk of postoperative anastomotic bleeding or leaks related to mechanical construction/perfusion issues. Potential advantages of this device warrant larger cohort studies and prospective randomized trials.

**Supplementary Information:**

The online version contains supplementary material available at 10.1007/s00268-023-06915-8.

## Introduction

The current colorectal anastomotic leak rate persists to be relatively high, broadly ranging between 5 and 15% [1]. The pathogenesis of anastomotic colorectal leaks is by all means multifactorial [2, 3]. Although the surgeon may have no power over the modification of tumor and patient-specific variables, surgical technique of anastomotic construction—encompassing lack of tension, adequate vascularity and appropriate application of basic principles of anastomosis creation—is instead directly controllable by the surgeon [1].

Several methods have been described for intraoperative evaluation of the integrity of colorectal anastomoses following left-sided colorectal resections. Technological evolution has allowed to progress from basic mechanical methods to the use of more sophisticated techniques, including oxygen spectroscopy, narrow-band imaging, Doppler flowmetry and fluorescence angiography [3].

The ideal method of anastomotic assessment should allow for a direct visualization of the anastomosis both intra- and extra-luminally, additionally combining the possibility of examining tissue microperfusion at this level.

This study describes a novel endoluminal modality of colorectal anastomotic assessment through the use of a rigid scope introducer also allowing for intraoperative perfusion evaluation by indocyanine green (ICG) fluoroangiography in patients undergoing laparoscopic left-sided colorectal resection.

## Materials and methods

**Fig. 1:**
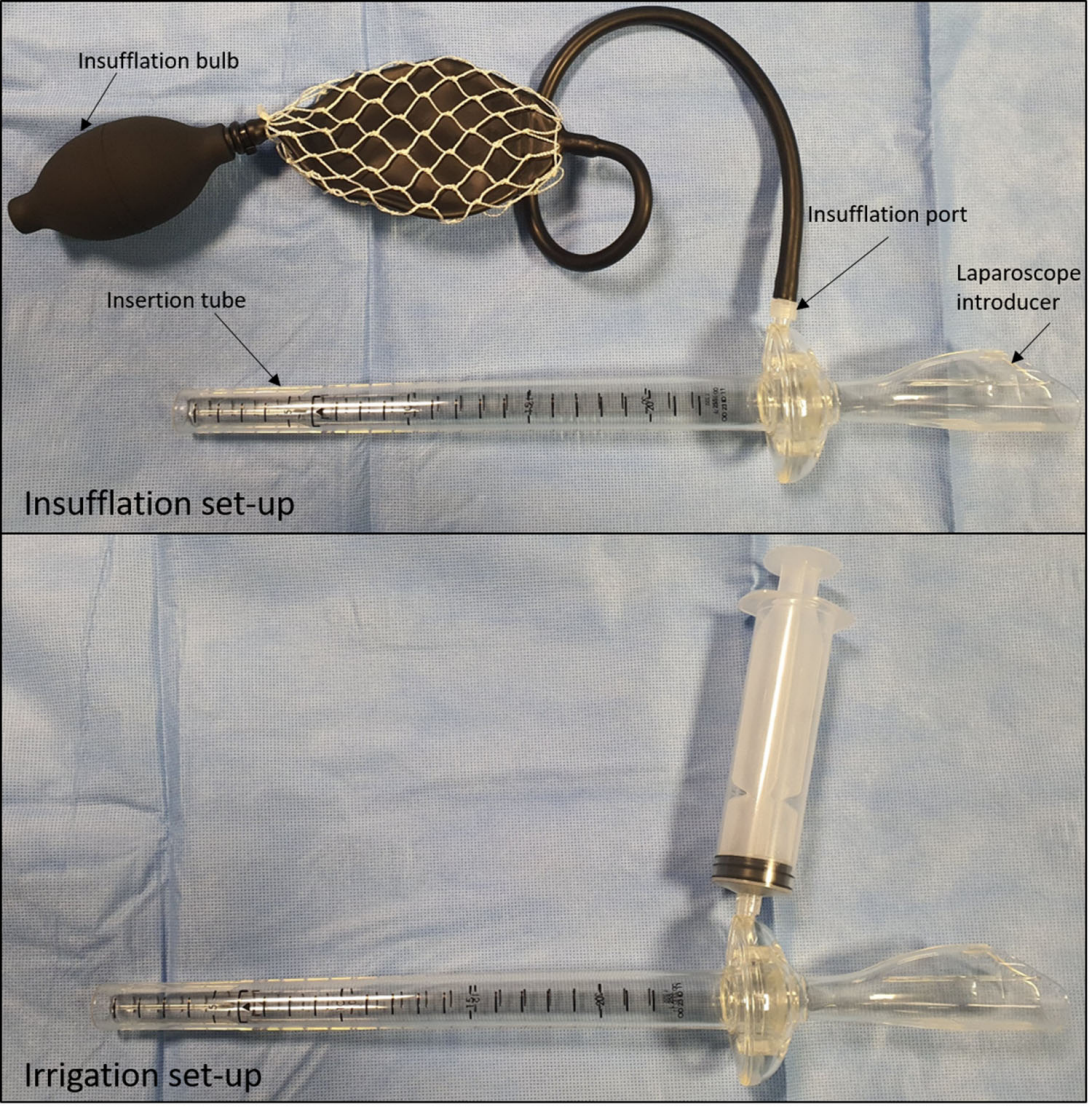
Disposable rigid scope introducer (DRSI) in insufflation (above) or irrigation setup (below). The DRSI can be connected to the insufflation bulb in order to adequately visualize the colorectal lumen or to a Luer-Lock syringe for irrigation/aspiration purposes

The US Food and Drug Administration (FDA)- and European Committee (EC)-approved Disposable Rigid Scope Introducer (DRSI) (Novadaq Technologies Inc., Canada) device is currently commercially available. The DRSI consists of an endoluminal introducer made up of an insertion tube and insufflation port (Fig. 1). The insertion tube length of the DRSI is 255 mm. A seal is positioned within the connection area between the insertion tube and the handle; the seal provides an airtight device valve for insufflation.

### DRSI setting

The DRSI is intended to be used in the rectum and distal portions of the colon. It is designed for use with a standard laparoscope, 320 mm in length, 10 mm in diameter, 0 or 30° lens.

Once the DRSI/laparoscope complex is assembled, it is inserted just past the anal verge and the rectum is inflated through the insufflation bulb. Under direct visual guidance of the white light image, the device is advanced to the level of the anastomosis which is assessed for staple line disruption, bleeding and fluoroangiographic microperfusion (Video S1).^1^

The ICG dose administered intravenously in the present study group was 0.3 mg/kg diluted in injectable sterile water. ICG injection was performed at two separate time points: at the moment of proximal colonic transection with direct visual inspection and after colorectal anastomotic construction with endoluminal assessment as evaluated through the DRSI device. An air-leak test was routinely performed while using the DRSI, by injecting saline solution in the abdominal field. (Additional technical details of the procedure are described in online Appendix.)

### Study design

This is a pilot prospective cohort single-center study designed to evaluate anastomotic integrity through the use of the DRSI with concomitant ICG fluoroangiography. Between January and March 2022, a total of 16 patients affected by colorectal cancer or diverticular sigmoid stricture underwent laparoscopic left colectomy, anterior resection or sigmoidectomy at Sapienza University Hospital in Rome, Italy. Demographics, comorbidities and history of neoadjuvant radiochemotherapy were also recorded and are summarized in Table 1.

**Table 1:** Patient demographics and characteristics

Patient	Gender	Age (years)	BMI (kg/m^2^)	Comorbidities	Previous abdominal surgery	Diagnosis	Neoadjuvant CHT/RT therapy
1	M	71	25.2	No	No	Adenocarcinoma of the mid rectum (6 cm from a.v.)	Yes
2	M	43	24	No	No	Diverticulitis	No
3	M	76	25.3	Hypertension	inguinal hernia repair	Descending colon adenocarcinoma	No
4	M	65	29	Hypertension; COPD	No	Sigmoid adenocarcinoma	No
5	M	72	25.6	Hypertension	No	Adenocarcinoma of the mid rectum (8 cm from a.v.)	Yes
6	F	69	28	Hypothyroidism	Histerectomy	Sigmoid diverticular stricture	No
7	M	72	24	Hypertension	No	Sigmoid adenocarcinoma	No
8	M	81	23	Hypertension	Appendectomy	Adenocarcinoma of the high rectum (11 cm from a.v.)	Yes
9	M	77	25	Type 2 diabetes	Open prostatectomy	Adenocarcinoma of the low rectum (4 cm from a.v.)	Yes
10	F	81	22.4	No	No	Descending colon adenocarcinoma	No
11	F	76	21	No	No	Sigmoid diverticular stricture	No
12	M	59	24.7	No	No	Sigmoid adenocarcinoma	No
13	M	68	22.1	No	No	Adenocarcinoma of the high rectum (12 cm from a.v.)	Yes
14	F	70	24.9	Hypertension	Left ovariectomy	Sigmoid adenocarcinoma	No
15	M	78	23.8	Type 2 diabetes	No	Adenocarcinoma of the mid rectum (9 cm from a.v.)	Yes
16	M	73	30.2	Hypertension; ischemic cardiomyopathy	No	Sigmoid adenocarcinoma	No

BMI: body mass index; CHT/RT: chemoradiotherapy; M: male; F: female; a.v.: anal verge; COPD: chronic obstructive pulmonary disease

At the moment of proximal colonic transection and after colorectal anastomotic construction, perfusion was evaluated and judged as either ‘excellent’ (intensely bright and uniform distribution of ICG at the designated level of proximal colon transection); ‘good’ (uniform distribution of ICG at the designated level of proximal colon transection), ‘poor’ (uneven distribution of ICG at the designated level of proximal colon) or ‘absent’ (no ICG detected). In case of ‘poor’ or ‘absent’ ICG perfusion, a change in transection point was made based on the area of greater fluorescence. A decision of avoiding loop ileostomies in low anterior resections (i.e., anastomosis ≤ 5 cm from anal verge), regardless of preoperative neoadjuvant radiochemotherapy, was made when a ‘good’ or ‘excellent’ anastomotic perfusion was visualized endoluminally.

Considering a mean time between injection and fluorescence visualization of an estimated 30–60 s and the ICG half-life of 150–180 s, we assumed 180 s as an upper time limit to assess fluorescence.

To prove the applicability of the DRSI device, we performed laparoscopic left-sided colorectal resections in 16 consecutive patients for malignant or benign conditions with endoluminal anastomotic integrity assessment. All surgical procedures and DRSI usage were performed by the same two surgeons. Institutional Review Board and Ethical Committee approval was obtained. All participants provided written informed consent to participate in the study and before all surgical procedures.

The Template for Intervention Description and Replication (TIDieR) guideline was used in the present study for intervention reporting purposes.

## Results

The DRSI was successfully used to assess anastomotic integrity after all left-sided colorectal resections performed in 16 consecutive patients (Table 1, 2).

**Table 2:** Perioperative data

Patient	Surgical procedure	Distance of anastomosis from anal verge (cm)	Endoluminal findings	ICG perfusion assessment	Intraoperative complications	Operative time (min)	Ileostomy	Clavien–Dindo	Postoperative hospital stay	30-day readmissions
1	LAR with TME	4	Patent, viable anastomosis	Excellent	No	210	No	–	6	No
2	Sigmoidectomy	15	Active anastomotic bleeding (12 o’clock)	Excellent	Anastomotic bleeding	198	No	–	5	No
3	Left hemicolectomy	12	Patent, viable anastomosis	Excellent	Perianastomotic serosal tear	260	No	–	5	No
4	Left hemicolectomy	10	Patent, viable anastomosis	Good	No	202	No	–	7	No
5	LAR with TME	4	Patent, viable anastomosis	Excellent	No	255	No	–	6	No
6	Sigmoidectomy	16	Patent, viable anastomosis	Good	No	178	No	–	6	No
7	Left hemicolectomy	11	Active anastomotic bleeding (1 o’clock)	Good	Anastomotic bleeding	191	No	–	5	No
8	AR with PME	8	Patent, viable anastomosis	Excellent	No	236	No	I	7	No
9	LAR with TME	2–3	Patent, viable anastomosis	Excellent	No	241	No	I	8	No
10	Left hemicolectomy	12	Patent, viable anastomosis	Excellent	No	211	No	–	5	No
11	Sigmoidectomy	16	Patent, viable anastomosis	Excellent	No	175	No	–	5	No
12	Left hemicolectomy	14	Patent, viable anastomosis	Good	No	188	No	–	6	No
13	AR with PME	7	Patent, viable anastomosis	Excellent	No	227	No	–	5	No
14	Left hemicolectomy	13	Patent, viable anastomosis	Excellent	No	236	No	I	7	No
15	LAR with TME	5	Patent, viable anastomosis	Good	No	240	No	–	6	No
16	Left hemicolectomy	12	Patent, viable anastomosis	Excellent	No	219	No	–	5	No

LAR: low anterior rectal resection; AR: anterior rectal resection; TME: total mesorectal excision; PME: partial mesorectal excision; ICG: indocyanine green

**Fig. 2:**
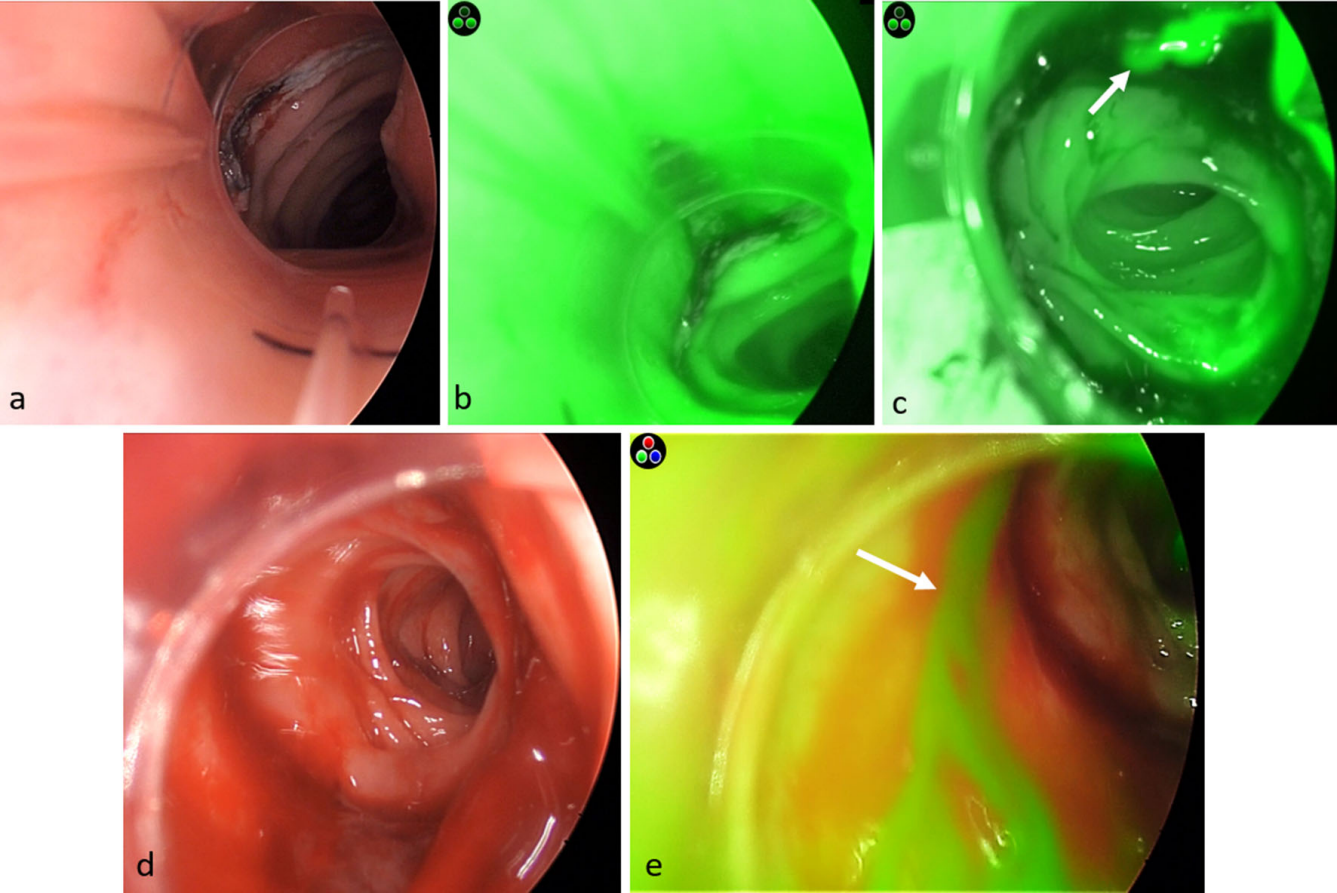
Representative endoluminal images obtained with the use of the DRSI and laparoscope with fluorescence imaging system. **a** Normal appearance of colorectal anastomosis in white light mode; **b** ICG fluoroangiography in grayscale mode showing ‘excellent’ level of perfusion; **c** evidence of active anastomotic bleeding at 12 o’clock (arrow) in grayscale mode; **d** presence of large amount of blood clots just below the anastomosis; **e** active bleeding in overlay fluorescence mode

All procedures were performed laparoscopically. No conversions to open surgery, major intra-/postoperative complications or mortality were recorded. Minor postoperative complications (Clavien–Dindo grade I) included one case of ileus, one wound infection and one urinary tract infection. The DRSI allowed to visualize the anastomotic site in all cases with a ‘good’ or ‘excellent’ quality of tissue perfusion by ICG fluoroangiography (Fig. 2). In two cases, the DRSI showed the presence of significant anastomotic bleeding which was successfully controlled by laparoscopic suture placement (Fig. 2) under direct endoluminal control with DRSI. Operative time was increased by an estimated 15 min compared to the same surgical procedures performed without the use of the DRSI. Perioperative outcomes are shown in Table 2.

## Discussion

Despite the substantial progress in technologies and refinement of surgical techniques, anastomotic leak rates still continue to complicate an elevated portion of colorectal procedures.

No definitive conclusions can yet be drawn from the current literature regarding the most effective modality of assessing anastomotic integrity [4]. A recent systematic review evaluating different approaches to colorectal anastomotic assessment could not find any superiority of one method over another, although advising to test anastomoses through basic air-leak tests to exclude mere mechanical failure [1].

An endoscopic view of the anastomosis allows for a more precise evaluation of tissue viability, as it has been proved that the mucosal layer has a lower tolerance to ischemia compared to the serosal lining [5]. The DRSI device allows for the concomitant use of a laparoscope and of an introducer combining the possibility of using a high-definition camera with a fluorescence imaging system for tissue perfusion assessment, at the same time conferring the possibility of adequate, albeit minimal, inflation and cleansing of the colorectal lumen for appropriate direct views of the colonic mucosa.

The DRSI demonstrated to have several advantages, including safety, limited costs, ease of use and high reproducibility. Additionally, it contributed to effectively indicate the presence of active anastomotic bleeding or adequate tissue perfusion in all cases. In our cohort of patients, the presence of good quality tissue perfusion visualized endoluminally via the DRSI drove the decision of avoiding loop ileostomies in selected cases. The limits we have experienced during the use of the DRSI include the need for an additional laparoscopic tower, the increase in the overall operative time, the possibility of exploring only up to 25 cm from the anal verge and the impossibility of performing operative maneuvers such as clip or suture placement when necessary (Table 3).

**Table 3:** Characteristics of the currently available intraoperative methods of anastomotic assessment with direct endoluminal visualization

	Intraoperative endoscopy	Transanal platform	DRSI
Controlled low-pressure insufflation			✓
Endoluminal ICG perfusion assessment		✓	✓
Visualization of high anastomoses	✓		✓ (up to 25 cm)
Non-traumatic for anal sphincter due to small diameter of instrument (< 2 cm)	✓		✓
Requires additional laparoscopic tower		✓	✓
Requires additional CO_2_ insufflator	✓	✓	
Allows operative maneuvers (eg. clip/suture placement)	✓	✓	
Short learning curve			✓
Low costs			✓
Time saving			✓

ICG: indocyanine green; DRSI: disposable rigid scope introducer

This device holds great potential for prompt intraoperative detection of anastomotic alterations, possibly reducing the risk of anastomotic leaks related to mechanical construction or perfusion issues. The potential advantages of this device warrant larger cohort studies and randomized trials.

## Supplementary Information

Below is the link to the electronic supplementary material.

Supplementary file1 (DOCX 38 KB)

## Footnotes

1 Video S1. Detailed use of the DRSI with a demonstration of four representative cases. The video can be found at: https://drive.google.com/file/d/1jGLHRatAJr20TBYULruc69sKwAI56K77/view?usp=sharing
