# Supplementary material for: Novel Modality of Endoluminal Anastomotic Integrity Assessment with Fluoroangiography After Left-sided Colorectal Resections
Source: World J Surg. 2023 Jan 24;47(5):1303–9. doi: 10.1007/s00268-023-06915-8 (PMC10070229; doi:10.1007/s00268-023-06915-8)
Supplement: Supplementary file 1 — Supplementary file1 (DOCX 38 KB) [file 268_2023_6915_MOESM1_ESM.docx]

**Appendix**

**Extended Methods**

The DRSI consists of an endoluminal introducer device made up of an insertion tube and insufflation port that is connected to a handle. The insertion tube length of the DRSI is 255 mm. The insertion tube is provided with graduated length markings in order to measure the distance from the anal verge. A seal is positioned within the connection area between the insertion tube and the handle; the seal provides an air-tight device valve for insufflation while the laparoscope is present within the DRSI. Additionally, an insufflation port enables attachment of a single-use insufflation bulb to manually insufflate the sigmoid and rectum (Figure 1).

*DRSI setting*

The DRSI is compatible with any laparoscopic camera, however, the use of a laparoscope equipped with a fluorescence imaging system is highly recommended in order to perform a fluoroangiography and assess perianatomotic perfusion.

Once the external surface of the DRSI has been lubricated, the insufflation bulb is connected to the Luer-lock port on the handle of the device. Once the DRSI/laparoscope complex is assembled it is inserted just past the anal verge and the rectum is inflated. Although a pressure value is not provided, the dimensions of the insufflation bulb is rather small (8 cm) and air capacity is relatively limited, hence allowing for an adequate but controlled distension of the colorectal lumen and of the anastomosis itself. Under direct visual guidance of the white light image provided by the laparoscope, the device is advanced to the level of the anastomosis which is assessed for the presence of staple line disruption and bleeding. If using a fluorescence imaging system, this would also provide the possibility to perform a fluoroangiography after the intravenous administration of ICG in order to check for any reduction of vascularity of the perianastomotic tissue. The ICG dose administered intravenously in the present study group was 0.3 mg/kg diluted in injectable sterile water.

*Fluorescence angiography*

Fluorescence imaging, which makes use of ICG’s fluorescent qualities, has grown in popularity over the past few years in a number of medical and surgical specialties. ICG is the fluorophore that is most commonly implemented in general surgery clinical practice due to its limited costs and wide availability [1-2].

ICG is a cyanine dye that is frequently used in medical diagnostics to assess hepatic function. Other employments of this fluorescent dye include perfusion assessment, lymph node navigation, extrahepatic biliary anatomy visualization, liver metastasis surgery, and plastic reconstructive surgery. The principle behind the ICG-fluorescent imaging method is that when protein-bound ICG is exposed to near-infrared light, it emits light with a peak wavelength of 830 nm.  The likelihood of anastomotic leakage correlates with intraoperative real-time perfusion assessment using ICG fluorescence imaging, highlighting the crucial link between adequate perfusion and anastomotic healing [1, 3].

*Surgical technique*

Patients were placed in a Lloyd-Davies position. All procedures were performed laparoscopically with a 4-trocar technique. A ligation of the inferior mesenteric vein and artery was performed at its origin. Dissection of the mesocolon from retroperitoneal structures was done in the avascular space between Toldt’s and Gerota’s fascia followed by the opening of the left colic gutter. Separation of the colo-epiploic ligament and full mobilization of the splenic flexure was routinely obtained. Transection of the colon or rectum, depending on the type of procedure, was done by using an endoscopic linear stapler with a blue cartridge, 45 or 60 mm in length. At this point, the specimen is extracted through a suprapubic incision. A first ICG injection (0.3 mg/kg) is given immediately before completing the resection to evaluate the area of fluorescence demarcation with the best perfusion. The anvil is placed in the proximal colon and secured with a purse string and placed back in the abdominal cavity. Pneumoperitoneum is reestablished and a colorectal anastomosis according to Knight-Griffen, using a circular 31 mm stapler, is completed. The DRSI is then used (as described in the Methods) to assess the anastomosis on the endoluminal side after a repeat ICG injection.

**Extended Discussion**

No definitive conclusions can yet be drawn from current literature regarding the most effective modality of assessing anastomotic integrity. Traditional Doppler ultrasonography, laser Doppler flowmetry, single-photon emission CT, mucosal oxygen saturation, and traditional angiography are some of the techniques used to assess blood flow. However, because the previously mentioned techniques have certain shortcomings, clinical judgment is typically used to assess the perfusion and viability of the surgically constructed colorectal anastomosis. Laser Doppler flowmetry, for instance, only detects microvascularity, because the pressure from the probe itself may affect local blood flow, laser Doppler flowmetry results cannot be replicated. Similarly, the other methods have a low reproducibility, are largely unavailable in regular operating rooms and have elevated costs [4].

In cardiac surgery and neurosurgery, ICG fluorescence angiography has been used to evaluate perfusion. Additionally, it has been demonstrated to be successful in esophageal and colorectal surgery for perfusion assessment purposes. The advantages of ICG fluorescence angiography include the ability to rapidly confirm blood flow, the possibility to measure blood flow repeatedly if necessary thanks to its short half-time (150 to 180 seconds), and the ability to detect both macrovascularity and microvascularity [5].

One of the main drawbacks of ICG fluorescence imaging is the lack of objectivity for evaluating tissue perfusion. In fact, at present, no software has been fully developed and widely or officially accepted for this purpose. Hence, ICG fluorescence can be currently judged purely based on subjective visual inspection. A limited number of studies were able to test and describe softwares which have the potential to measure quantitative ICG fluorescence at fixed points during gastric tube construction after esophagectomy [4-6]. Authors conclude how these technologies provide potential parameters in fluorescence imaging signal quantification that could aid the surgeon in assessing perfusion [4, 6]. Nevertheless, there is still a need for larger-scale investigations to confirm such interesting findings.

A prospective multicenter single-arm study, the PILLAR II trial, examined the feasibility and safety of performing fluorescence angiography for perfusion assessment before colorectal transection and after completing the anastomosis [7]. Although performing a similar method of endoluminally evaluating the anastomosis to the one illustrated in the present study, a thorough description of how this was done was not included. We provide a full technical description of this method (Video S1).

A recent meta-analysis assessed the effect of using ICG fluoro-angiography in patients undergoing colorectal surgery and compared them to patients who had only direct visual inspection of the anastomosis. Authors concluded that ICG angiography evaluation of colorectal anastomoses is possibly related with a decreased risk of anastomotic leak than conventional white light evaluation. However, a higher likelihood of anastomotic leak may be linked to changes in surgical plan based on ICG fluoro-angiography [8].

An endoscopic view of the anastomosis allows for a more precise evaluation of tissue viability, as it has been proven that the mucosal layer has a lower tolerance to ischemia compared to the serosal lining [9]. Hence, the endoscopic assessment can consent to directly observe areas of underperfusion of the colonic mucosa. The intraoperative endoluminal examination of a colorectal anastomosis is commonly performed with the use of sigmoidoscopes or rectoscopes. However, such methods do not permit to evaluate accurate tissue perfusion. On the contrary, modern laparoscopes offer several advantages such as higher definition cameras in addition to a fluorescence imaging system allowing to evaluate perianastomotic tissue vascularity. Simple laparoscopes, nevertheless, lack some practical features which are necessary for endoluminal examination, such as the possibility of insufflating the distal colon or rectum as well as suction-irrigation systems to facilitate proper visualization of the mucosal surface. The DRSI device allows for the concomitant use of a laparoscope and of an introducer combining the possibility of using a high definition camera with a fluorescence imaging system for tissue perfusion assessment, and at the same time conferring the possibility of adequate inflation and cleansing of the colorectal lumen for appropriate direct views of the colonic mucosa.

**References**

1. Mangano A, Masrur MA, Bustos R, Chen LL, Fernandes E, Giulianotti PC. Near-Infrared Indocyanine Green-Enhanced Fluorescence and Minimally Invasive Colorectal Surgery: Review of the Literature. Surg Technol Int. 2018 Nov 11;33:77-83.
2. Baiocchi GL, Diana M, Boni L. Indocyanine green-based fluorescence imaging in visceral and hepatobiliary and pancreatic surgery: State of the art and future directions. World J Gastroenterol. 2018 Jul 21;24(27):2921-2930. doi: 10.3748/wjg.v24.i27.2921.
3. Langer D, Vočka M, Kalvach J, Ryska M. Assessment of anastomosis perfusion by fluorescent angiography in robotic low rectal resection: the results of a non-randomized study. Rozhl Chir. 2019 Winter;98(3):110-114.
4. Jansen SM, de Bruin DM, Wilk LS, van Berge Henegouwen MI, Strackee SD, Gisbertz SS, van Bavel ET, van Leeuwen TG. Quantitative Fluorescence Imaging of Perfusion-An Algorithm to Predict Anastomotic Leakage. Life (Basel). 2022 Feb 8;12(2):249. doi: 10.3390/life12020249.
5. Pacheco PE, Hill SM, Henriques SM, Paulsen JK, Anderson RC. The novel use of intraoperative laser-induced fluorescence of indocyanine green tissue angiography for evaluation of the gastric conduit in esophageal reconstructive surgery. Am J Surg. 2013 Mar;205(3):349-52; discussion 352-3. doi: 10.1016/j.amjsurg.2012.11.005.
6. Yukaya T, Saeki H, Kasagi Y, Nakashima Y, Ando K, Imamura Y, Ohgaki K, Oki E, Morita M, Maehara Y. Indocyanine Green Fluorescence Angiography for Quantitative Evaluation of Gastric Tube Perfusion in Patients Undergoing Esophagectomy. J Am Coll Surg. 2015 Aug;221(2):e37-42. doi: 10.1016/j.jamcollsurg.2015.04.022.
7. Jafari MD, Wexner SD, Martz JE, McLemore EC, Margolin DA, Sherwinter DA, Lee SW, Senagore AJ, Phelan MJ, Stamos MJ. (2015) Perfusion assessment in laparoscopic left-sided/anterior resection (PILLAR II): a multi-institutional study. J Am Coll Surg. Jan;220(1):82-92.e1. doi: 10.1016/j.jamcollsurg.2014.09.015.
8. Emile SH, Khan SM, Wexner SD. Impact of change in the surgical plan based on indocyanine green fluorescence angiography on the rates of colorectal anastomotic leak: a systematic review and meta-analysis. Surg Endosc. 2022 Apr;36(4):2245-2257. doi: 10.1007/s00464-021-08973-2.
9. Daya B Singh, Gerard Stansby, Iain Bain, David K Harrison. Intraoperative measurement of colonic oxygenation during bowel resection. (2009) Adv Exp Med Biol. 645:261-6. doi: 10.1007/978-0-387-85998-9_39.

**
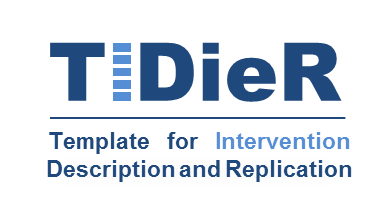
The TIDieR (Template for Intervention Description and Replication) Checklist*:**

| **Item number** | **Item** | **Where located **** | |
| --- | --- | --- | --- |
|  |  | Primary paper  (page or appendix  number) | Other ^†^ (details) |
|  | **BRIEF NAME** |  |  |
| **1.** | Provide the name or a phrase that describes the intervention. | ____1, 4______ | ______________ |
|  | **WHY** |  |  |
| **2.** | Describe any rationale, theory, or goal of the elements essential to the intervention. | ____3, 4______ | _____________ |
|  | **WHAT** |  |  |
| **3.** | Materials: Describe any physical or informational materials used in the intervention, including those provided to participants or used in intervention delivery or in training of intervention providers. Provide information on where the materials can be accessed (e.g. online appendix, URL). | 4-6, 9 Video S1 | Appendix pg. 1-2 |
| **4.** | Procedures: Describe each of the procedures, activities, and/or processes used in the intervention, including any enabling or support activities. | ____4-6______ | _____________ |
|  | **WHO PROVIDED** |  |  |
| **5.** | For each category of intervention provider (e.g. psychologist, nursing assistant), describe their expertise, background and any specific training given. | ____5-6______ | Appendix pg. 1-2 |
|  | **HOW** |  |  |
| **6.** | Describe the modes of delivery (e.g. face-to-face or by some other mechanism, such as internet or telephone) of the intervention and whether it was provided individually or in a group. | ____4-6______ | Appendix pg. 1-2 |
|  | **WHERE** |  |  |
| **7.** | Describe the type(s) of location(s) where the intervention occurred, including any necessary infrastructure or relevant features. | _____5_______ | _____________ |
|  | **WHEN and HOW MUCH** |  |  |
| **8.** | Describe the number of times the intervention was delivered and over what period of time including the number of sessions, their schedule, and their duration, intensity or dose. | __5-6, Table 2_ | Appendix pg. 1-2 |
|  | **TAILORING** |  |  |
| **9.** | If the intervention was planned to be personalised, titrated or adapted, then describe what, why, when, and how. | _____5-6_____ | Appendix pg. 1-2 |
|  | **MODIFICATIONS** |  |  |
| **10.^ǂ^** | If the intervention was modified during the course of the study, describe the changes (what, why, when, and how). | _____N/A_____ | _____________ |
|  | **HOW WELL** |  |  |
| **11.** | Planned: If intervention adherence or fidelity was assessed, describe how and by whom, and if any strategies were used to maintain or improve fidelity, describe them. | _____ N/A ____ | _____________ |
| **12.^ǂ^** | Actual: If intervention adherence or fidelity was assessed, describe the extent to which the intervention was delivered as planned. | ______6-7____ | _____________ |

** **Authors** - use N/A if an item is not applicable for the intervention being described.

† If the information is not provided in the primary paper, give details of where this information is available. This may include locations such as a published protocol or other published papers (provide citation details) or a website (provide the URL).

ǂ If completing the TIDieR checklist for a protocol, these items are not relevant to the protocol and cannot be described until the study is complete.

* We strongly recommend using this checklist in conjunction with the TIDieR guide (see *BMJ* 2014;348:g1687) which contains an explanation and elaboration for each item.

* The focus of TIDieR is on reporting details of the intervention elements (and where relevant, comparison elements) of a study. Other elements and methodological features of studies are covered by other reporting statements and checklists and have not been duplicated as part of the TIDieR checklist. When a **randomised trial** is being reported, the TIDieR checklist should be used in conjunction with the CONSORT statement (see [www.consort-statement.org](http://www.consort-statement.org)) as an extension of **Item 5 of the CONSORT 2010 Statement.** When a **clinical trial** **protocol** is being reported, the TIDieR checklist should be used in conjunction with the SPIRIT statement as an extension of **Item 11 of the SPIRIT 2013 Statement** (see [www.spirit-statement.org](http://www.spirit-statement.org)). For alternate study designs, TIDieR can be used in conjunction with the appropriate checklist for that study design (see [www.equator-network.org](http://www.equator-network.org)).
